# Dynamic X-ray Microtomography vs. Laser-Doppler Vibrometry: A Comparative Study

**DOI:** 10.21203/rs.3.rs-4874430/v1

**Published:** 2024-08-08

**Authors:** Aleksandra Ivanovic, Jeffrey Tao Cheng, Margaux Schmeltz, Christian M. Schlepütz, Anne Bonnin, Lukas Anschuetz

**Affiliations:** Department of Otorhinolaryngology, Head and Neck Surgery, Inselspital, Bern University Hospital, University of Bern, Switzerland, 2Hearing Research Laboratory, ARTORG Center for Biomedical Engineering Research, University of Bern, Switzerland, Paul Scherrer Institut, Swiss Light Source, Villigen PSI, Switzerland,; Department of Otolaryngology, Head and Neck Surgery, Mass. Eye and Ear, Boston Children Hospital, Harvard Medical School, Boston, 02114, MA, USA; Swiss Light Source, Paul Scherrer Institut, Villigen PSI, Switzerland; Swiss Light Source, Paul Scherrer Institut, Villigen PSI, Switzerland; Swiss Light Source, Paul Scherrer Institut, Villigen PSI, Switzerland; Department of Otorhinolaryngology, Head and Neck Surgery, CHUV Centre Hospitalier Universitaire Vaudois and University of Lausanne, Lausanne, Switzerland

**Keywords:** Middle ear, laser-Doppler vibrometry, dynamic synchrotron-based X-ray microtomography, phase-contrast

## Abstract

**Purpose:**

There are challenges in understanding the biomechanics of the human middle ear, and established methods for studying this system show significant limitations. In this study, we evaluate a novel dynamic imaging technique based on synchrotron X-ray microtomography designed to assess the biomechanical properties of the human middle ear by comparing it to laser-Doppler vibrometry (LDV).

**Methods:**

We examined three fresh-frozen temporal bones (TB) using dynamic synchrotron-based X-ray microtomography for 256 Hz and 512 Hz, stimulated at 110 dB and 120 dB SPL. In addition, we performed measurements on these TBs using 1D LDV, a well-established method.

**Results:**

The normalized displacement values (μm/Pa) at the umbo and the posterior crus of the stapes are consistent or within 5–10 dB differences between all LDV and dynamic microtomography measurements and previously reported literature references. In general, the overall behavior is similar between the two measurement techniques.

**Conclusion:**

In conclusion, our results demonstrate the suitability of dynamic synchrotron-based X-ray microtomography in studying the middle ear’s biomechanics. However, this study shows that better standardization regarding acoustic stimulation and measurement points is needed to better compare the two measurement techniques.

## 1 Introduction

The middle ear is a complicated and delicate biomechanical system that plays a vital role in hearing. It is located within the temporal bone and hosts the three auditory ossicles, malleus, incus, and stapes, which are connected to ligaments and attached to two muscles in the air-filled tympanic cavity. Over the past century, research has deepened our understanding of its mechanics [1–4]. The middle ear’s primary function is to amplify and transmit air-borne sound waves to the fluid-filled cochlea. Impedance matching plays a crucial role: when sound waves strike the eardrum, the auditory ossicles work together to compensate for the impedance mismatch between air and inner ear fluid. This process results in a nearly 50-fold (*≈* 34 dB increase) amplification of force transmitted to the inner ear. Additionally, the tensor tympani and stapedius muscles modulate the ossicles’ movement. Disruptions in the ossicular chain can lead to conductive hearing loss, while its total absence results in a hearing loss of approximately 60 dB sound pressure level (SPL) [5].

However, studying the middle ear’s biomechanics is challenging for several reasons: The middle ear is embedded into the temporal bone, and visual access to all three ossicles is limited. In addition, the movements are very tiny. The stapes footplate moves approximately 10 nm at a stimulus of 80 dB SPL [6].

Several measurement techniques have been proposed to study the dynamic behavior of the middle ear ossicles. Laser-Doppler vibrometry (LDV), which uses the Doppler effect to measure vibrations, has been employed as the gold standard. The interaction of two coherent laser beams, a reference, and a target beam, is used to calculate the beams’ movement toward the target structure. Displacements less than 10^−8^ mm and frequencies between 100 Hz and 100 kHz can be measured. The LDV measurements are single-point measurements, which provide information only in the direction of the beam. To evaluate the surface motion of an area, scanning LDV with multiple measurements at the selected location is conducted [7–9].

Nevertheless, this measurement approach comes with certain drawbacks. Firstly, LDV measurements require reflective beads to be placed manually in the region of interest, and the alignment of the laser beam to the beads can be complicated and impedes easy reproduction of the experimental results. Additionally, the experimental setup is complex and requires continuous calibrations. Another point is the required direct microscopic sight of the area of interest. To access the stapes, one must remove some anatomical structures of the auditory system (opening of the facial recess and sectioning of the stapedius muscle) to get direct sight. However, this only allows access to limited parts of the crura, the neck, and the head of the stapes, excluding the footplate. In addition, a one-directional LDV is traditionally used to resolve stapes motions along its piston-like direction. However, 3D motions of the ossicles have gained more and more interest in recent years, which requires the adaption of a 3D LDV [10–12].

Various other measurement techniques, such as holography [13–15] and optical coherence tomography [16–19], have proven helpful in understanding the dynamic behavior of the tympanic membrane and the ossicular chain. However, both these techniques are only sensitive to the movement along the pathway of the optical beam. Thus, none of these measurement techniques allow us to simultaneously visualize and quantify the dynamic behavior of the ossicular chain in 3D.

An imaging method developed in recent years that can overcome some of the limitations mentioned above is dynamic synchrotron-based microtomography. In addition to laboratory or clinical micro-CT scanners [20, 21], static synchrotron-based X-ray microtomography has allowed new morphological studies of the inner and middle ear. By harnessing the unique properties of synchrotron radiation, this technique provides enhanced spatial resolution and soft tissue contrast by exploiting phase contrast in addition to absorption contrast, making it ideal for investigating delicate anatomical structures without destruction [22–24]. More recently, we were also able to perform dynamic synchrotron-based X-ray microtomography for the first time on unstained fresh-frozen human ear specimen and extract the three-dimensional information of the dynamic behavior of the auditory ossicles during sound stimulation of 110 dB and 120 dB SPL at 128 Hz [25]. To correlate our recently proposed dynamic imaging method for evaluating the biomechanics of the human middle ear, we compared it to LDV measurements on the same temporal bone (TB) specimen. This paper presents a quantitative comparison of the two techniques.

## 2 Methods

### 2.1 Ethical approval

The study protocol was approved by the local ethical committee of Bern (Kantonale Ethikkommission Bern, KEK-BE 2016–00887) and the local ethical committee of the Paul Scherrer Institute (Ethikkommission Nordwest-und Zentralschweiz, 2017–00805), as well as the Mass General Brigham Institutional Review Board (#2022P001306).

### 2.2 Dynamic synchrotron-based X-ray microtomography

Three fresh-frozen human TBs anonymous donors were provided by the Eaton Peabody Laboratories, Mass Eye and Ear, Boston, MA, USA. The donor of TB1 was a 51-year-old white male (right ear), the donor of TB2 was an 89-year-old black female (right ear), and the donor of TB3 was a 58-year-old white male (left ear). They are stored as *B-Fresh1*, *B-Fresh2*, and *B-Fresh3* in the PSI petabyte archive system (a tape-based long-term storage system at the Swiss National Supercomputing Centre CSCS in Lugano, Switzerland). For simplicity, we have changed the naming of the samples in this article. TB1 corresponds to the raw data of *B-Fresh1*, TB2 corresponds to the raw data of *B-Fresh2*, and TB3 corresponds to the raw data of *B-Fresh3*. For better readability, we will refer to dynamic synchrotron-based-phase contrast X-ray microtomography simply as dynamic microtomography henceforth.

#### 2.2.1 Sample preparation

The three fresh-frozen TBs were dissected as follows: Laterally, the concha was removed, conserving the bony and cartilaginous external auditory canal. Posteriorly and superiorly, the air cells of the mastoid portion were removed entirely until the tegmen tympani and the antrum. Inferiorly, the soft tissue was removed until the internal carotid artery, the jugular bulb, and the insertion of the Eustachian tube. Medially, the petrous part of the temporal bone was removed until the bony capsule of the labyrinth. The semicircular canals and the internal auditory canal were skeletonized. Finally, the sample included an intact external auditory canal, middle and inner ear, and had a size of approximately 5 × 2 cm. The surrounding temporal bone was reduced to a maximum thickness of 1 mm to minimize the X-ray absorption.

**Figure 1:**
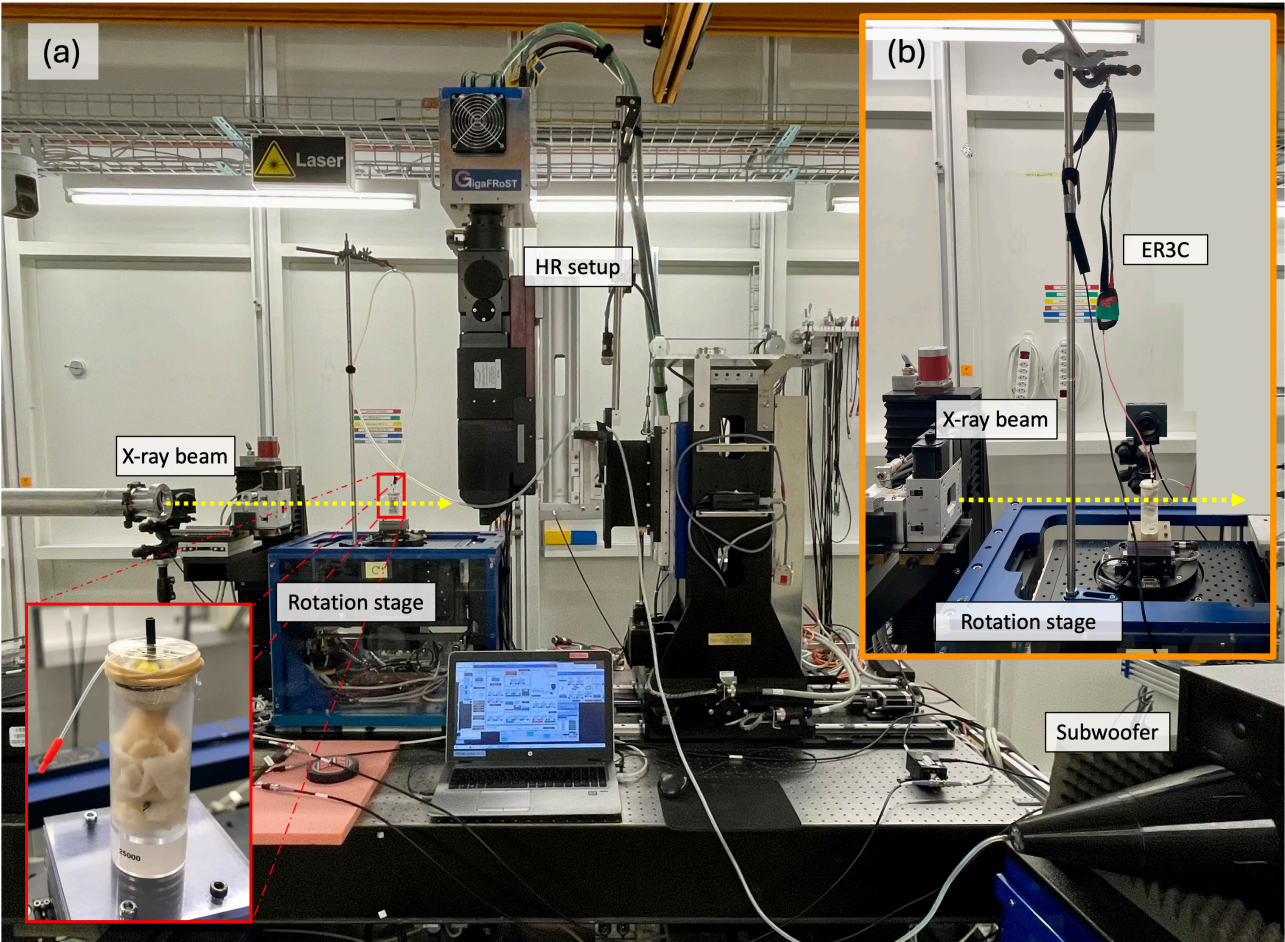
Experimental setup at the TOMCAT beamline. The sample is wrapped in saline-soaked neuro-patties to keep it from drying, fixed in a custom-made sample holder, and mounted onto the rotation stage. The top is sealed with a plastic film, showing two tubes coming out (see zoom-in, bottom left of (a)). The black one is the tip of the earplug, which is sewn on one end to the external ear canal and connected over a silicone tube to the subwoofer (a) or to ER3C Insert Earphones from Etymotic coupled to an amplifier (b) on the other hand. The transparent tube with the red tip is inserted into the probe microphone during calibration. The sample is fixed onto the rotation stage. The X-ray beam comes from the left (indicated with a yellow arrow). The HR setup consists of the in-house built GigaFRoST camera [26] and a 4x magnification high numerical aperture macroscope from Optique Peter [27].

An earplug was sewn into the external auditory canal. During the image acquisition, the specimen was placed in a custom-made cylindrical holder (diameter of 25 mm) and mounted on the rotation stage at the TOMCAT beamline. To prevent the samples from drying out during the acquisition, they were wrapped in neuro-patties soaked in a sterile saline solution, and the top of the holder was sealed with a plastic film (see Figure 1).

#### 2.2.2 Sound stimulation and calibration

Before scanning each sample, we calibrated the sound stimulation with a clinical probe microphone (ER7C, Etymotic Research) by measuring the exact voltage we needed to apply to the auditory canal to reach the desired dB SPL at a particular frequency. We measured at 256 Hz and 512 Hz with 110 dB and 120 dB SPL. We used a sub-woofer with an inverted cone attached for 256 Hz and ER3C Insert Earphones from Etymotic coupled to an amplifier for 512 Hz and connected either of them to a sine wave generator (MeasComp USB daq Module MC1608 USB-1608G SKU: 6069-410-059). A silicon tube connected the sound stimulation unit to the earplug we sewed to the external auditory canal.

#### 2.2.3 Image acquisition and reconstruction

Dynamic synchrotron-based X-ray phase-contrast microtomography was conducted at the TOMCAT beamline (X02DA) within the Swiss Light Source (Paul Scherrer Institute, Switzerland). A multi-scale strategy was implemented to accommodate the dimensions of human TBs. Initially, a low-resolution (LR) setup was employed to capture overview scans of the sample, followed by local high-resolution (HR) scans of the middle ear. An in-house developed Fiji plugin, utilizing the 3D reconstructed LR dataset as input, facilitated the determination of spatial coordinates for regions of interest to be imaged with the HR setup [28]. The LR overview scans covered a field-of-view (FOV) of approximately 29 × 12.5 mm^2^, using a half-acquisition technique, which entails a 360° rotation rather than the standard 180° in tomography acquisitions. The setup comprised a PCO 5.5 Edge camera coupled with a 1:1 microscope positioned 3 meters from the sample, resulting in an effective pixel size of 5.8 μm. Scan parameters were adjusted to minimize radiation exposure, with a 30 ms exposure time and 1000 projections spanning 360°.

The dynamic HR acquisitions were performed with a custom-made in-house fast read-out system consisting of the GigaFRoST camera [26], a LuAg:Ce scintillator with a thickness of 150 μm, and a 4x magnification high numerical aperture macroscope from Optique Peter [27]. These components were configured at a propagation distance of 250 mm, yielding an effective pixel size of 2.75 μm [27]. The FOV achieved was approximately 11 × 3.3 mm² using the ”half-acquisition” method. LR and HR acquisitions used a polychromatic beam filtered with a 5 mm Sigradur and a 4 mm glass filter. Additional filtration for LR acquisitions included a 15 mm Sigradur and a 75 μm Molybdean filter to minimize sample dose. The resulting average energy was approximately 24 keV.

Given the assumption of periodic vibration in the middle ear, with a frequency matching that of the sound stimulation, each motion cycle occurs much more rapidly than the time required for a complete set of angular projections in tomography acquisition (which typically entails several thousand images). To accommodate this, dynamic tomograms were constructed by gathering a substantial number of projections across multiple consecutive motion cycles while the rotation stage slowly rotated. A total of 40,000 projections were captured during a single 360° rotation for each scan, which took 20 seconds at 256 Hz, and 28 seconds at 512 Hz.

While keeping the maximum FOV, the maximum frame rate of the GigaFRoST camera is at 2 kHz before saturating the data transfer. This corresponded to a minimum exposure period of 0.5 ms between consecutive image acquisitions. Consequently, the exposure period was always maintained above 0.5 ms to prevent saturation of the read-out system while maintaining a consistent FOV across all frequencies. The exposure time, i.e., the effective photon collection duration, was adjusted based on the frequency of sound stimulation. It always stayed within one-tenth of the sound stimulation period to prevent image blurring due to motion. Thus, exposure time decreased with increasing sound stimulation frequency, ranging from 0.3 ms for 256 Hz to 0.19 ms for 512 Hz.

To correct for the X-ray beam inhomogeneities and dark current of the camera, the projections were first dark- and flat-field corrected. The sinograms were then computed for each set of projections and then reconstructed using the filtered back-projection Gridrec algorithm [29] and the Sarepy algorithm for ring removal [30].

Two signals were collected during the image acquisition: the sinusoidal signal (or gating signal) transmitted from the signal generator to the sound unit and the camera exposure signal, giving the exact time of each image acquisition. These two signals allowed us to associate each image with a specific phase of the sine stimulation, corresponding to a specific phase of the vibration of the middle ear. The gating signal period was decomposed into ten different time windows called phases $p_{j}$, with $p_{0}$ being the reference phase taken at the ascending zero-crossing point of the sinusoidal curve. A post-gating algorithm was applied to the 40,000 raw projections to sort them into the correct phases and build ten post-gated tomograms of approximately 4000 projections. These 4000 projections were evenly distributed over the full 360° rotation of the sample, so that each post-gated tomogram provided a 3D reconstruction of each specific phase of the middle ear motion cycle.

#### 2.2.4 Data analysis

A detailed description of the analysis pipeline is given by Schmeltz & Ivanovic et al.[25]. Note that the pipeline developed to analyze the dynamic synchrotron-based X-ray microtomography data extracts the motion in all three directions. To allow for a more accurate comparison of the two techniques, we adapted the dynamic microtomography pipeline to also extract displacements in only one direction. We tried to match the direction in which the motion was extracted as closely as possible to the direction in which the LDV measurements were taken. For the umbo, this is along the direction perpendicular to the plane of the tympanic annulus. For the stapes, it is perpendicular to the stapes footplate. This allows for a more accurate comparison between the two measurement techniques.

To assess the movement of the ossicular chain in response to sound stimulation, we assumed that the ossicles act as independent rigid bodies. According to this presumption, their three-dimensional motion over time can be characterized by rigid transformations composed of a rotation followed by a translation, i.e., all points within a given ossicle undergo identical transformations. Therefore, analyzing only a subset volume (SV) of an ossicle is sufficient to deduce the transformation of the entire ossicle. As previously mentioned, each motion cycle was divided into ten distinct time intervals anointed phases $p_{j}$. The intensity-based registration algorithm **imregtform** from Matlab was employed to perform a 3D registration of the SV of an ossicle imaged at phase $p_{0}$ with the corresponding SV imaged at the other phases $p_{j}$, where j∈ [1,9] to estimate the geometric transformation aligning the two phases without the need of segmentation or manual placement of landmarks. It effectively uses all available information by considering the unaltered intensity of every image pixel, thereby enabling sub-voxel registration [31]. Three SVs were manually selected for each ossicle. The transformations of all SVs were then averaged to obtain an average transformation per phase for each ossicle.

After obtaining the mean transformations for all phases $p_{j}$ across the three ossicles, the sinusoidal displacement of any region of interest (ROI) within an ossicle could be determined by calculating the projection of the displacement vector in z-direction applied to that point. To compare to the LDV measurement points, two ROIs (the umbo and the posterior crus of the stapes) were manually chosen using Fiji from the reconstructed data stack captured at phase $p_{0}$. To assess the precision of displacement computation compared to manual ROI selection, five points were selected around the ROI to compute the standard deviation of displacement estimations.

We applied the pipeline to a portion of the petrous bone to ensure that the extracted transformations corresponded to vibrations of the stimulated ossicles and not to vibrations of the entire sample in the sample holder. These values set the noise limit for our analyses.

### 2.3 Laser-Doppler Vibrometer

#### 2.3.1 Sample preparation

The identical three specimens that underwent dynamic microtomography were refrozen and returned to Eaton Peabody Laboratories, Mass Eye and Ear, Boston, MA, USA. Further preparation of the temporal bones consisted of opening the facial recess to confirm the normality of the middle ear structures and to gain access to the stapes for laser-Doppler vibrometry (LDV) measurements [32]. A small part of the anterior-superior wall was opened and later replaced by a transparent plastic window to allow LDV measurement of umbo displacement (see Figure 3).

#### 2.3.2 Laser measurements

Small retro-reflective tape pieces (approximately 100 μm × 100 μm × 60 μm thick) were affixed to the lateral surface of the tympanic membrane (TM) at the umbo and to the posterior crus of the stapes [8]. Each sample was securely positioned on an air-isolation table within a soundproof booth. LDV measurements were initially conducted at the umbo and then transitioned to the stapes without altering the setup. Additionally, vibrations of the petrous bone near the oval window were recorded to assess the noise floor and stimulus artifact in the measured displacements; all of the umbo and stapes motion measurements we report are at least 20 dB above the driven vibration of the petrous bone. For sound stimulation, a speaker (Radio Shack) equipped with a plastic tube was tightly sealed to the opening of the ear canal to deliver sound to the external ear. At the same time, a calibrated probe microphone (PCB 377C10) monitored sound pressures in the ear canal $\left({\text{P}}_{ec}\right)$ within a distance of less than 2 mm from the TM surface. The hardware of the stimulus and recording system, as well as its software control, have been detailed previously by Ravicz and Rosowski, 2012 [33]. The primary stimulus was a sequence of 50 pure tones with frequencies logarithmically spaced between 200 and 20,000 Hz, during which the stimulus voltage to the loudspeaker remained constant at 0.5 V.

#### 2.3.3 Data analysis

Fourier transforms of the recorded microphone and LDV time waveforms described the complex (magnitude and phase angle) sinusoidal sound pressure and velocity at the stimulus frequency. The velocities were converted into displacements by dividing the complex velocity by (2π×frequency×i) and then normalized by the complex sound pressure at the stimulus frequency $\left({\text{P}}_{ec}\right)$. We report the normalized displacement magnitude by the sound pressure for a single stimulus of 0.5 V. In addition, we cosine corrected the displacement for the angle of the measuring beam to account for the expected piston-like stapes movement of the footplate.

## 3 Results

Dynamic synchrotron-based X-ray microtomography scans were conducted on three fresh frozen temporal bones (TB1–3) at two intensities, 110 dB and 120 dB SPL, and two frequencies, 256 Hz and 512 Hz. The measurements were conducted at the TOMCAT beamline at the Swiss Light Source (Paul Scherrer Institute, Switzer- land). After the dynamic imaging scans, the three TBs were shipped to the Eaton Peabody Laboratories, Mass Eye and Ear, Boston, MA, USA, where they underwent LDV measurements at the umbo, the posterior crus of the stapes, and the petrous bone. The acoustic stimulus of 0.5 V was sent to the speaker, which generated frequency-dependent sound between 80 dB and 100 dB SPL for a frequency range of approximately 200 – 20000 Hz.

### 3.1 Dynamic synchrotron-based X-ray microtomography

We normalized the unidirectional displacement amplitudes to the ear canal pressure $P_{ec}(\text{μm}/\text{Pa})$ to compare the two results quantitatively. Table 1 shows the raw displacement amplitudes (μm) and the normalized displacement amplitudes (μm/Pa) for the umbo and the posterior crus of the stapes for the three TBs at the two intensities and frequencies. For better comparison, 110 dB SPL is denoted as 6.89 Pa and 120 dB SPL as 21.79 Pa. We see that umbo and stapes displacement increase with increasing stimulus levels. However, the normalized displacement values decrease with increasing intensity except for TB1 and TB3 measured at the umbo at 256 Hz.

For example, Figure 4 shows the mean rotation angle and translation magnitude for TB1 stimulated at 512 Hz and 120 dB SPL. It confirms that the movement we extract for the malleus (a) is sine-like and periodic. In comparison, (b) depicts the extraction from the same temporal bone stimulated at 512 Hz and 110 dB SPL, where we can not define a sine-like behavior for the rotation angle; it shows the registration detected from the moving background texture (noise). Because the algorithm can not detect any moving feature, we see higher ”artificial rotation.” Fortunately, this background movement does not have the periodicity of sound stimulation, which lets us discriminate the artificial movement from the actual movement. Similar behavior was found for TB2 and TB3 at 512 Hz and TB1 at 256 Hz, all stimulated at 110 dB SPL, which indicates that the actual movement (if any) is too small at this sound intensity stimulation to be detected by the algorithm. The displacements denoted in Table 1 represent the average of the ”artificial displacement” detected by the algorithm.

### 3.2 Laser- Doppler Vibrometry

The TBs were measured with LDV at three points of interest (POI): the umbo, the posterior crus of the stapes, and the petrous bone, with pure tones at a constant stimulus level of 0.5 V delivered to the speaker, sweeping through frequencies between 200 – 20000 Hz. Figure 5 and Figure 6 show the normalized displacement magnitude (μm/Pa) of the LDV measurements versus frequency at the umbo and posterior crus of the stapes, respectively. In addition, the normalized displacement values from the dynamic microtomography measurements at 120 dB SPL for the three TBs at 256 Hz and 512 Hz are marked with green, blue, and pink crosses, respectively. As a reference, values from Gan et al. (2004)[8] and Goode et al. (1994)[34] are plotted in red and black, respectively. At the umbo, we can observe a variance between the LDV measurements of the three TBs within the normal range [8, 34]. However, for the measurements taken at the posterior crus of the stapes, the LDV measurements are generally lower than the reference values from Gan et al. [8]. However, the values from the measurements taken at the synchrotron are much closer to the reference data values analyzed at the posterior crus of the stapes. In general, we can see that all values acquired at the synchrotron are within the normal range of previously reported LDV measurements and either consistent or within 5–10 dB differences between all LDV measurements taken at the same samples.

## 4 Discussion

This study compared dynamic synchrotron-based X-ray microtomography to laser- Doppler vibrometry to assess middle ear biomechanics in three temporal bone samples. Our results showed either consistent or within 5–10 dB differences between the normalized displacement values for the umbo and the posterior crus of the stapes in the LDV technique compared to those displacement values measured with dynamic synchrotron-based X-ray microtomography, as seen in Figure 5 and 6.

During the dynamic microtomography scans, we stimulated the sample with a constant sound pressure of 110 and 120 dB SPL by adjusting the voltage to the corresponding frequency. In comparison, during the LDV scans, we sent a constant voltage of 0.5 V to the speaker, and therefore, the pressure in the ear canal changed depending on the frequency. Although the stimulation strategies used differ, we observe similar SPL values for the two frequencies we looked at. However, a difference of 5–10 dB of overall displacements was observed between the two techniques. The discrepancy between the two measurement techniques is less likely caused by different stimulus levels. However, the dynamic microtomography scans were performed before the LDV. The samples underwent thawing, high radiation, and refreezing before they were shipped to Boston for the LDV measurements. This could have led to the drying of the soft tissue components in the bones. Moreover, opening the facial recess and sectioning of the stapedius muscle for the LDV measurements could have also affected the samples. Nevertheless, the general behavior for the umbo and the stapes is consistent between the dynamic microtomography and the LDV measurements, comparable with previously reported data, and within the measured frequency range.

Table 1 shows a decrease in displacement amplitude from the umbo to the stapes, which is expected from previous literature. To ensure that the extracted transformations corresponded to vibrations of the stimulated ossicles and not to external motion (such as vibrations of the whole system), we applied the analysis pipeline to parts of the petrous bone. We found that the algorithm registers the textured, moving background (noise) when there is no detectable motion in the structure. This background noise varies between images, depending, amongst others, on the sample size, the exposure time (correlated to the frequency), the camera calibrations, and the scintillator used. This leads to different limits of our system to detect the smallest possible movement, depending on the acquisition. However, these movements do not show any sine-like behavior, which allows us to discriminate them from the stimuli-induced movement and, therefore, verify the transformations we extract on the ossicles.

The normalized dynamic microtomography displacement amplitudes (μm/Pa) measured at 110 dB SPL (6.89 Pa) are generally higher than those measured at 120 dB SPL (21.79 Pa) for all TB and frequencies. This is indicated in Table 1. An increase in 10 dB SPL in a linear regime corresponds to a displacement ratio of 3.16. However, we observe a maximum ratio of 2.47 at the umbo and 2.92 at the stapes, corresponding to an increase of 7.86 dB and 9.31 dB, respectively. This either suggests compressive growth - a slower growth in displacement than the stimulus level - for both the umbo and stapes, or it outlines our technical limitations for dynamic synchrotron X-ray microtomography, mentioned above. There are several reports on nonlinear behavior in the middle ear for high sound pressure level stimulus [35–37]. Recently, Cheng et al. reported that the onset of nonlinear growth depends on the sample and can start at 110 dB SPL [36]. They reported mainly expansive growth at the umbo for frequencies below 2 kHz and compressive growth at the stapes for frequencies below 2 kHz, which would only partially align with our findings. In addition, since we do not have measurements acquired with a stimulus below 110 dB SPL, we can not state whether we are already in a nonlinear regime at 110 dB SPL (continuous exposition to 6.89 Pa). Another explanation would be our technical limitations in resolving displacements at a sub-pixel level.

**Figure 2:**
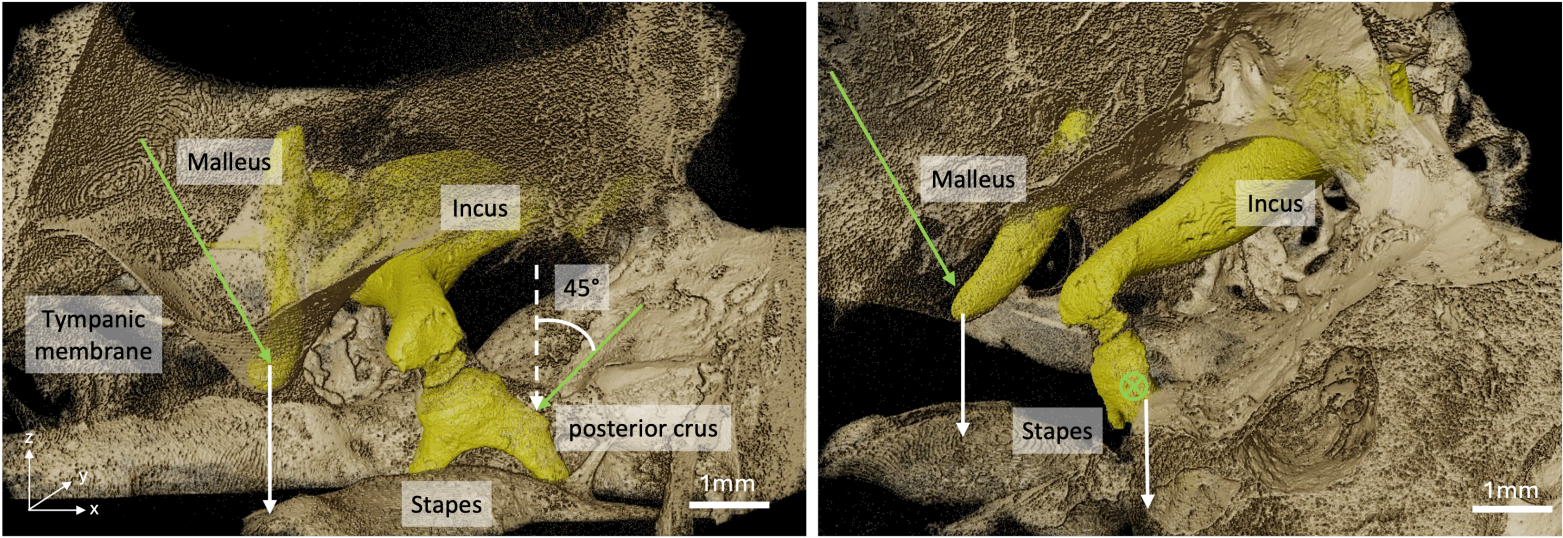
Direction of displacement extraction. The ossicles are displayed in yellow, surrounded by petrous bone, and the tympanic membrane in gold. White arrows indicate the directions of movement extraction from the dynamic microtomography data (in z-direction). Green arrows approximate the direction of the laser beam. As indicated in the figure, for this TB, we measured at the posterior crus of the stapes at an angle of 45°. We can then apply a cosine correction to derive the stapes motion along the z-direction, aligned with the stapes footplate. We can not approximate the angle properly for the umbo; therefore, the direction difference between the LDV and dynamic microtomography seems to be more extensive.

**Figure 3:**
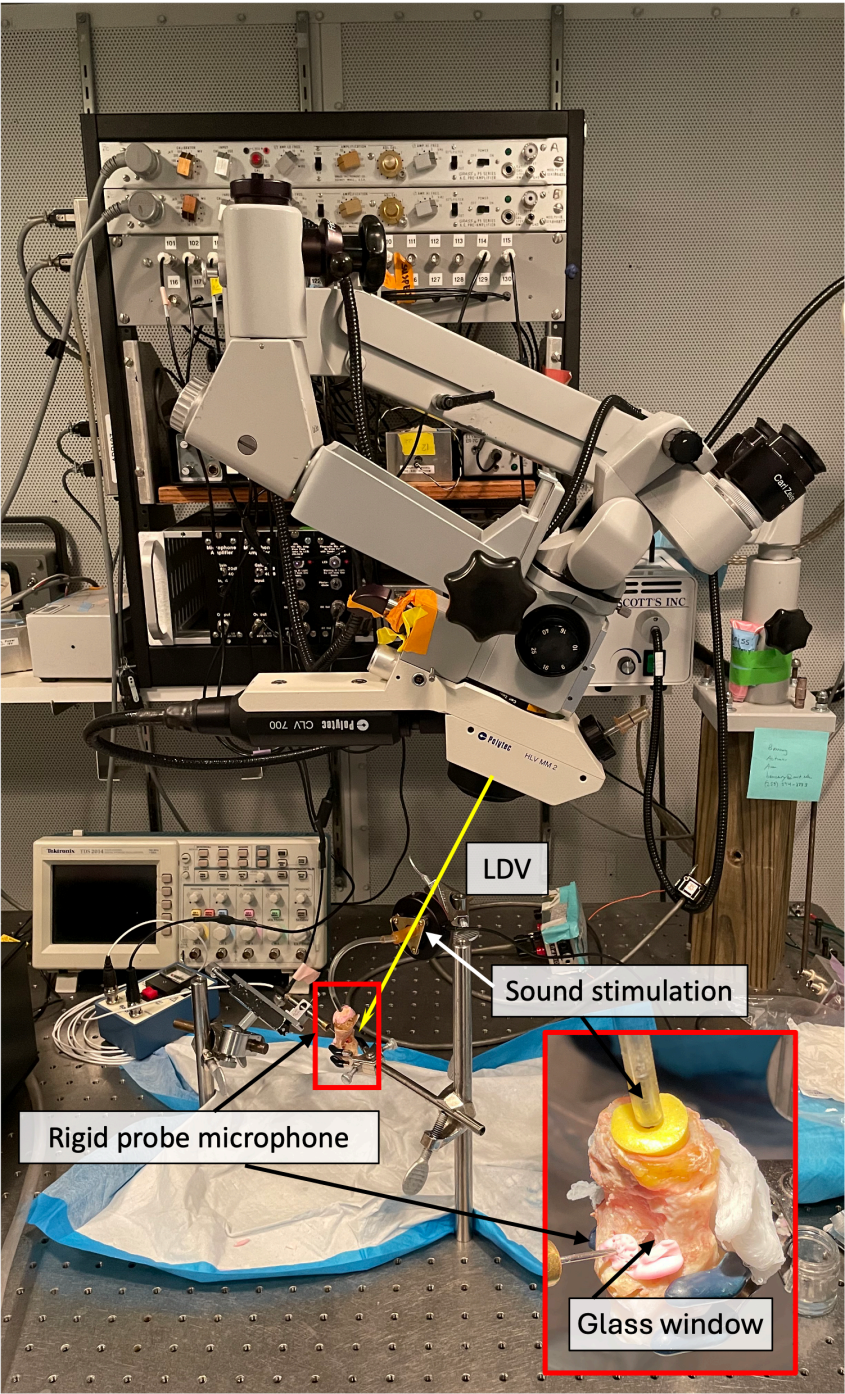
Experimental LDV setup at the Eaton Peabody Laboratories. The sample is fixed on an air-isolation table within a soundproof booth. Part of the ear canal wall is replaced by a transparent plastic window that allows LDV measurement of the umbo. Reflective beads are placed at the area of interest (umbo, posterior crus of the stapes, and petrous bone). In addition, a probe microphone is inserted into the ear canal through a hole in the plastic window to monitor ear canal sound pressure (Pec) levels near the tympanic membrane (TM). LDV measurement of the stapes displacement is performed through the glass-covered opening in the facial recess (see zoom-in on the bottom right). We connected the sound stimulation to the yellow earplug, similar to the dynamic microtomography measurements. The laser beam (indicated with a yellow arrow) is manually focused on the reflective beads previously placed in the area of interest.

**Figure 4:**
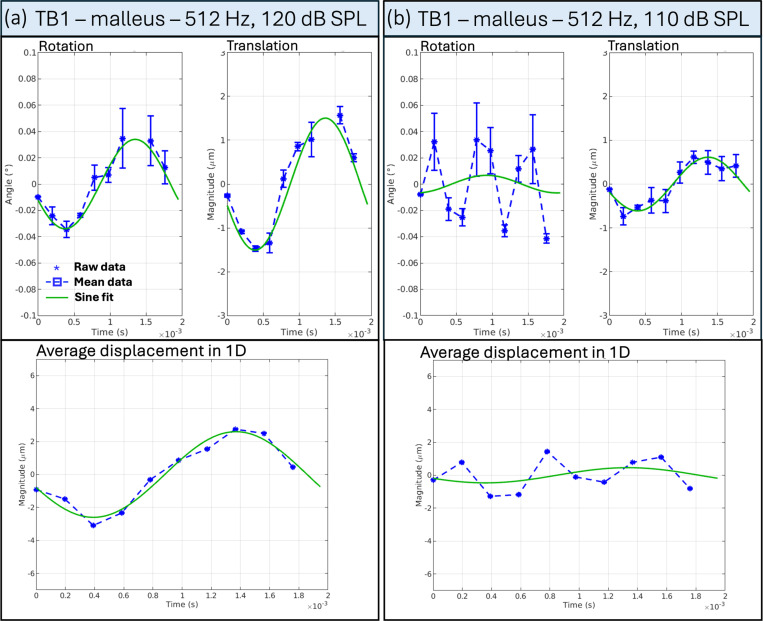
Motion quantification for the malleus of TB1 stimulated at 512 Hz, for the two intensities, 110 dB and 120 dB SPL. (a) shows the mean rotation angle and the mean translation magnitude following a sine behavior for TB1 stimulated at 512 Hz and 120 dB SPL computed for the umbo. The computed average displacement in 1D similarly shows the sine-like behavior. (b) shows the same TB1 stimulated at 512 Hz and 110 dB SPL. We can see the ”artificial rotation” detected by the algorithm caused by the moving textured background because the feature displaces no actual movement or a movement that is too small to be registered. Accordingly, the average displacement in 1D shows no sine-like behavior.

**Figure 5:**
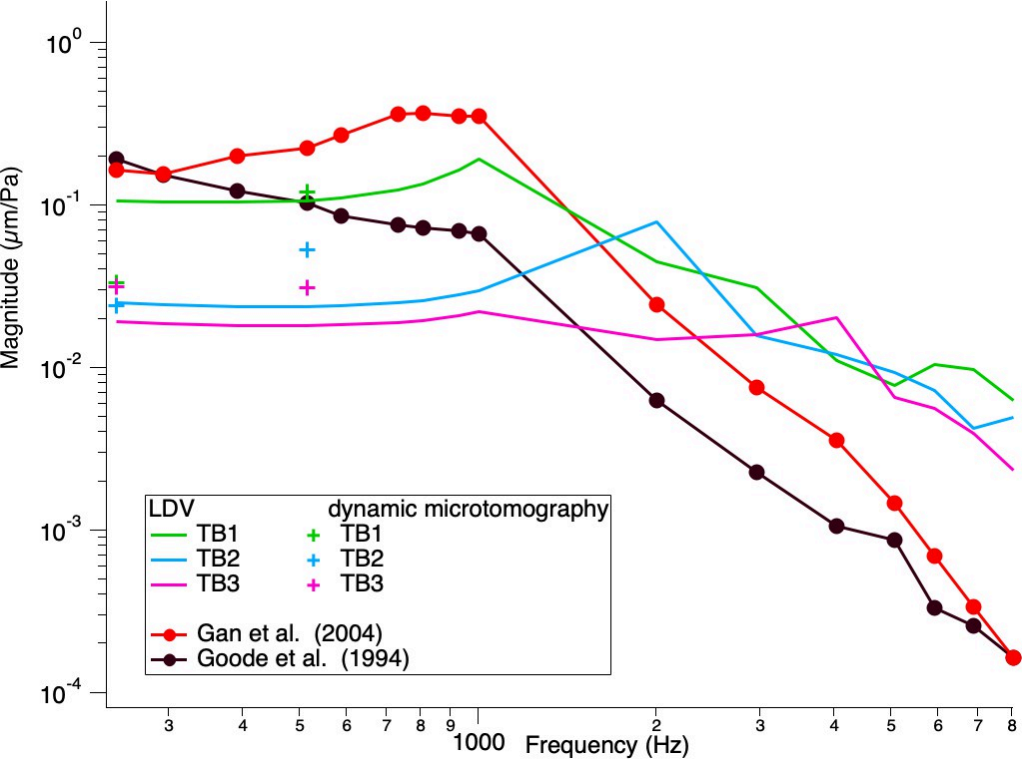
Umbo normalized displacement magnitude (μm/Pa) vs frequency. Comparison of our LDV measurements at the umbo with dynamic microtomography measurements at 256 and 512 Hz and previous literature. All measurements are within a normal range and comparable to previous literature values. There is a steeper slope in displacement between 256 Hz and 512 Hz for the dynamic microtomography data of TB1 and TB2 compared to our LDV data, similar to the data from Gan et al. (2004). TB3 shows a similar plateau compared to our LDV data.

**Figure 6:**
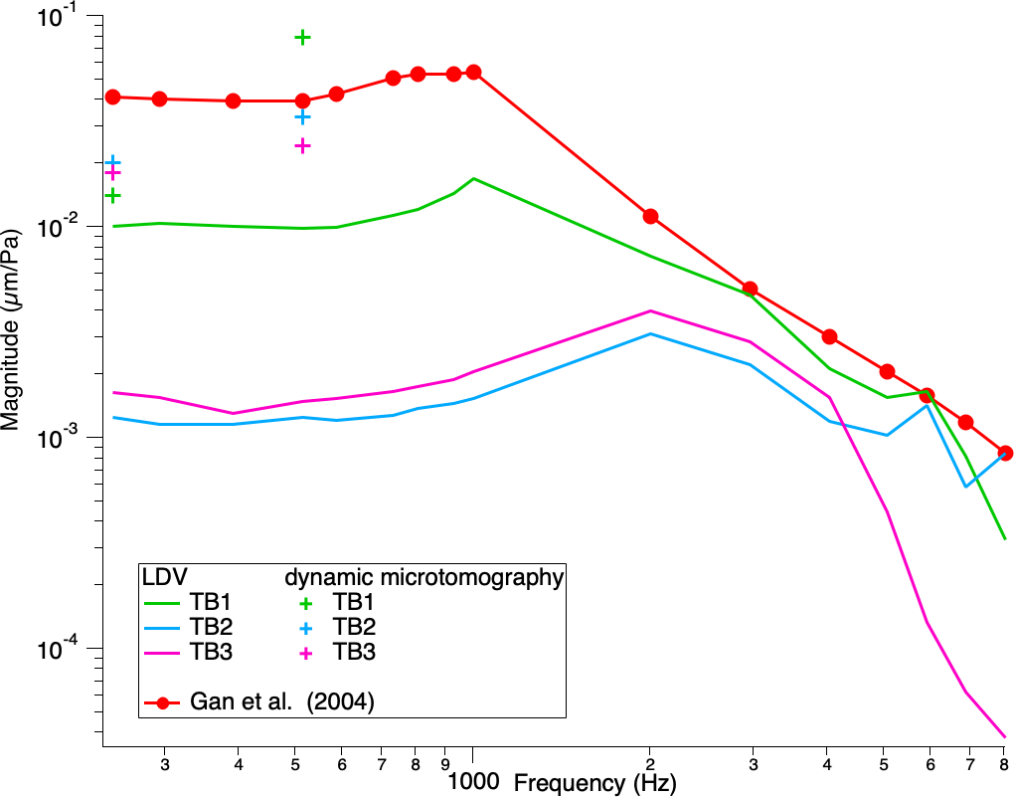
Stapes posterior crus normalized displacement magnitude (μm/Pa) vs frequency. Comparison of our LDV measurements at the stapes posterior crus with dynamic microtomography measurements at 256 and 512 Hz and previous literature. We can see an increase in displacement from 256 Hz and 512 Hz for all TBs. TB1 shows the steepest increase, while TB2 and TB3 are flatter. All LDV measurements are notably lower than the literature reference and the dynamic microtomography measurements.

Comparing two measurement techniques is not straightforward. With 1D LDV, we measure the velocity of a moving object in a single direction only, along the laser beam. The laser is manually focused on the point of interest for each sample. However, the anatomy of each specimen is different. This makes it impossible to align the laser beam along the same axis between different TBs. However, laser focusing is usually done by the same experienced person for different samples during an experiment. Therefore, we can suggest that the setup and sample orientation are as similar as possible, considering the difference in the individual anatomy between the three TBs. Similarly, one person prepared all TBs for the dynamic microtomography measurements and placed them in the sample holder in the same orientation (external auditory canal facing upwards) (see Figure 1). To mimic the 1D measurement from LDV, we extracted the displacement in z-direction only for the dynamic microtomography data analysis, as illustrated in Figure 2 for one sample. However, we recently showed that the movement of the umbo and the stapes is not just unidirectional and only piston-like [25], but more complicated already at low frequencies, stimulated at 120 dB SPL. Unfortunately, we could not estimate the angle of the LDV measurements at the umbo compared to the direction we extracted from the dynamic microtomography because we did not have the images reconstructed when we acquired the LDV measurements. We do not have pictures of every sample from which we could estimate the laser beam exactly enough. Consequently, one can never capture the same direction along the movement in one direction only.

Furthermore, in the dynamic microtomography measurements, we are mainly limited by the photon flux provided by the Swiss Light Source, which is required for a short exposure time while maintaining good image quality. The acquisition parameters are tied to the limited photon flux for dynamic microtomography: a trade-off between frame rate and FOV must be made to achieve sufficient acquisition speed. However, with the upgrade of the Swiss Light Source, the upgrade of the TOMCAT beam-line, and the new generation of high-numerical aperture microscope, we can expect to reach a voxel size of at least 1.8 μm with a better image quality, opening the door to measurements at higher stimulation frequencies.

In the future, it would be interesting to compare the entire six degrees of freedom of the ossicles by comparing 3D LDV measurements with dynamic microtomography. Conducting the LDV measurements first, followed by the dynamic microtomography, might help reduce possible sample changes due to radiation. However, this would mean that the sample would no longer be intact for the dynamic microtomography scans. Further, one could consider keeping the sound stimulation during the dynamic microtomography at a constant voltage instead of a sound pressure level to have more comparable stimulation intensity values. In addition, with the ongoing upgrade of the Swiss Light Source, we will be able to increase the spatial and temporal resolution and, therefore, might be able to resolve movements at frequencies up to 2kHz. This would allow a comparison on a broader frequency range.

## Conclusion

Upon assessing dynamic synchrotron-based X-ray microtomography and quantitatively comparing it to the gold standard technique, laser-Doppler vibrometry, we found

that the former proves to be a suitable imaging technique for studying the middle ear’s biomechanics.

However, this study shows that better standardization is needed to compare the two measurement techniques. It is particularly challenging to compare the two methods quantitatively because, in 1D LDV, we look at single-point measurements, while with dynamic microtomography, we can extract the transformation matrix for each ossicle and apply it to any point of interest.

## Figures and Tables

**Table 1:** Raw and normalized displacement amplitudes. Raw and normalized displacement amplitudes measured at the umbo and the posterior crus of the stapes, for 6.89 and 21.79 Pa, 256 and 512 Hz, and the three TBs. There is an increase in all raw displacement amplitudes from 6.89 Pa to 21.79 Pa. The normalized values show a more variable behavior. TB1 generally shows the highest raw displacements compared to TB2 and TB3, which show more similar values.

**ROI**	umbo							
Pressure (Pa)	6.89				21.79			
Frequency (Hz)	256		512		256		512	
**displacement**	raw disp	norm disp	raw disp	norm disp	raw disp	norm disp	draw disp	norm disp
TB1	0.345	0.050	1.236	0.179	0.721	0.720	2.590	0.033
TB2	0.289	0.042	0.745	0.108	0.521	0.024	1.148	0.053
TB3	0.278	0.040	0.271	0.039	0.677	0.310	0.670	0.031
**ROI**	posterior crus stapes							
Pressure (Pa)	6.89				21.79			
Frequency (Hz)	256		512		256		512	
**displacement**	raw disp	norm disp	raw disp	norm disp	raw disp	norm disp	raw disp	norm disp
TB1	0.13	0.019	0.95	0.138	0.31	0.014	1.73	0.080
TB2	0.21	0.030	0.25	0.036	0.44	0.020	0.73	0.034
TB3	0.14	0.020	0.27	0.039	0.40	0.018	0.53	0.024
